# Effects of texture properties of semi-solid food on the sensory test for pharyngeal swallowing effort in the older adults

**DOI:** 10.1186/s12877-020-01890-4

**Published:** 2020-11-23

**Authors:** Jin-Woo Park, Seul Lee, Byoungseung Yoo, Kiyeon Nam

**Affiliations:** 1 Department of Physical Medicine and Rehabilitation, Dongguk University Ilsan Hospital, 27 Dongguk-ro, Ilsandong-gu, Goyang-si, Gyeonggi-do 10326 Republic of Korea; 2 Department of Food and Nutrition Service, Dongguk University Ilsan Hospital, Goyang-si, Gyeonggi-do Republic of Korea; 3 Department of Food Science and Biotechnology, Dongguk University, Goyang-si, Gyeonggi-do Republic of Korea

**Keywords:** Dysphagia, Diet, Viscosity, Texture, Ageing

## Abstract

**Background:**

Increasing viscosity can reduce the risk of aspiration into the airway, but excessively thickened food may require more force and effort. We assumed that semi-solid foods with similar viscosities will behave differently in the oropharynx and there might exist the possibility that properties other than viscosity may have clinical relevance. This study aimed to find out the texture of semi-solid foods that affects the effort of pharyngeal swallow in the older adults.

**Methods:**

Nine kinds of semi-solid foods not requiring mastication were selected for texture profile analysis (TPA), and included whipped cream, mayonnaise, soft tofu, mango pudding, boiled mashed pumpkin, boiled mashed potatoes, boiled mashed sweet potatoes, red bean paste, and peanut butter. Hardness, adhesiveness and cohesiveness of each food were measured three times by using the rheometer. A blinded sensory test using a 9-point hedonic scale was also conducted in eighteen older adults people to investigate how much effort was required to swallow food, and how much of the food remained in the pharynx after swallowing. The correlation between texture and sensory outcome was statistically analyzed.

**Results:**

Foods that belonged to the same viscosity category showed different texture values, and the participants also rated different scores respectively. Only adhesiveness among three properties was significantly correlated with the sensory test. (*r* = 0.882, *p* = 0.002 for difficult to swallow, *r* = 0.879, *p* = 0.002 for sense of residue).

**Conclusions:**

Adhesiveness was the most important property of the semi-solid foods, requiring most efforts in pharyngeal swallow in the older adults. If we select and provide food having low adhesiveness value in the same viscosity category, there might be the possibility to make it easier to swallow in older adults.

**Supplementary Information:**

The online version contains supplementary material available at 10.1186/s12877-020-01890-4.

## Background

Oropharyngeal dysphagia refers to having difficulty in transporting food safely from the mouth to the esophagus. It can cause dehydration, nutrition deficit, aspiration pneumonia and even death [1, 2]. It commonly occurs in patients with neurologic deterioration such as stroke or Parkinson disease [3] or head and neck cancers [4]. Because the incidence of dysphagia-related diseases increases with age, dysphagia occurs more frequently in the older adults [5].

Clinically, the use of viscosity-modified food has become very important in the treatment of dysphagia [6–8]. It is widely known that the liquid like water flows quickly and can pose a risk to people with dysphagia [6, 9]. Therefore, thickened liquids are highly recommended as slowing down the flow rate can provide the time required to close the airways [6, 10]. In contrary, excessively thickened food may require much more force on the tongue and pharynx during swallowing. People with weakened tongue and pharyngeal muscles are at risk of leaving residues in the pharyngeal recess after swallowing [11, 12].

However, we have experienced the effect of semi-solid foods with similar viscosity on swallowing is not the same. Mostly, it requires a lot of effort, but sometimes it is easy and safe for example, peanut butter vs. soft tofu. Which factors make this difference? We assumed that food texture (hardness, adhesiveness or cohesiveness), other than viscosity, might have clinical relevance. Hardness is the force that is required to compress food between the tongue and palate to a given deformation or to penetration. Adhesiveness is defined that the work required to remove food that adheres to the mouth (generally the palate) during the normal swallowing process and cohesiveness means the strength of internal bonds making up the body of the food [13]. It was especially thought to affect older adults or dysphagia patients than healthy adults. Therefore, this study was aimed to find out the texture of semi-solid food affects the effort of pharyngeal swallow in the older adults.

## Methods

### Test foods selection

Nine kinds of semi-solid foods with similar viscosities (fork test [14] grade 1, IDDSI [15] level 4 category and British Dietetic Association [16] Texture C) not requiring mastication were chosen for this experiment. They included whipped cream, mayonnaise, soft tofu, mango pudding, boiled mashed pumpkin, boiled mashed potatoes, boiled mashed sweet potatoes, red bean paste, and peanut butter. They were all commercially available at the market as finished products.

### Texture profile analysis (TPA)

**Fig. 1:**
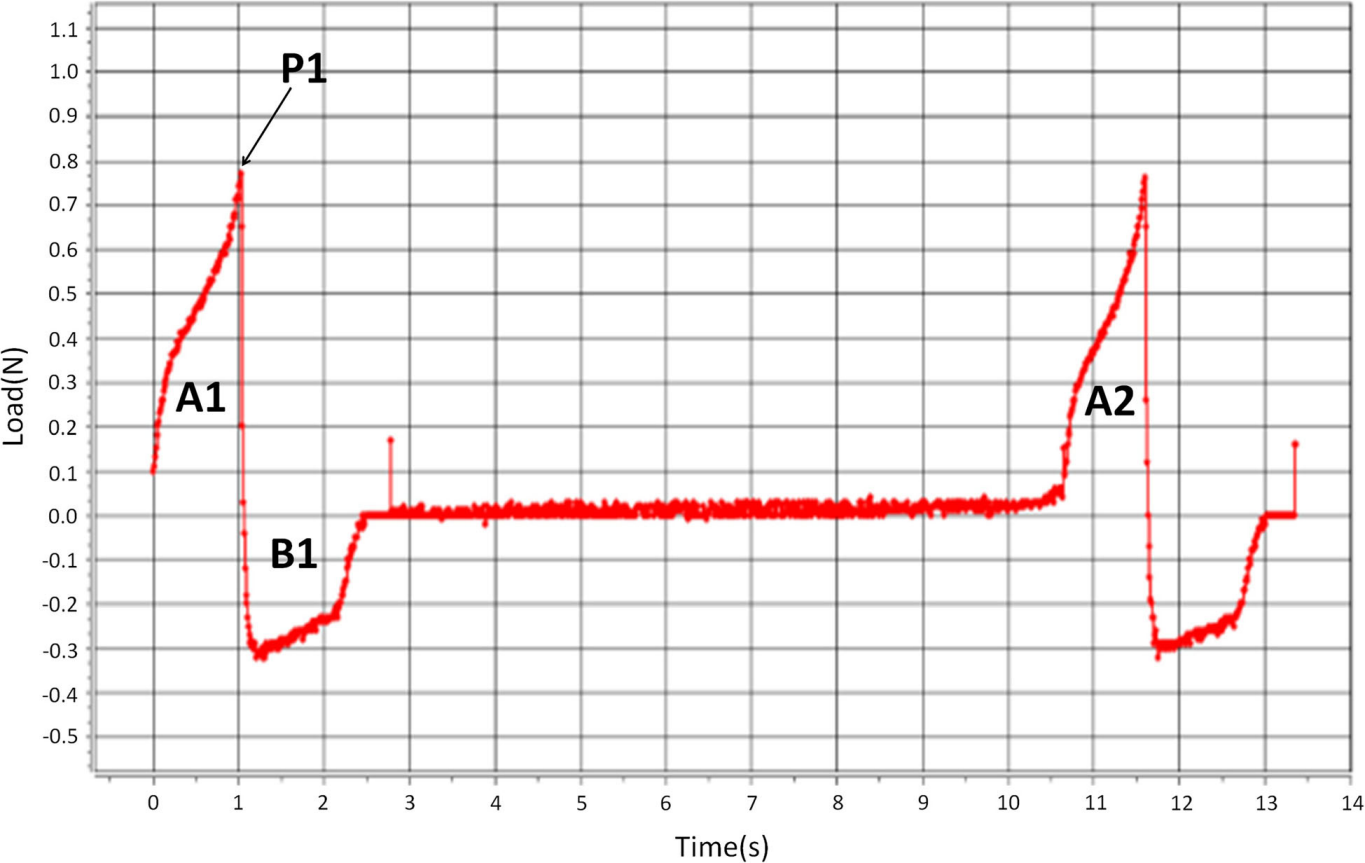
Parameter of texture profile analysis. Hardness; P1, adhesiveness; B1, cohesiveness; A2/A1

We measured the hardness, adhesiveness and cohesiveness of each food three times immediately before performing sensory tests by using a CT3 texture analyzer (AMETEK Brookfield, MA, USA) (central temperature 20 ± 0.2 °C, stain 70%, probe diameter 20 mm, infiltration meter 10.5 mm, test speed 10 mm/s). The texture profile parameters (Fig. 1) were determined as follows: (1) hardness was defined as the maximum force required for compressing foods and was calculated as the peak force of the first compression (P1) (2) adhesiveness was calculated as the negative area (B1) for the first bite, representing the work necessary to pull the compressing plunger away from the sample (3) cohesiveness was calculated as A2/A1 (A1 and A2 represent the integrated energy required for the first and second compression, respectively).

### Sensory test

#### Subjects

Eighteen healthy older volunteers (two males and sixteen females), with an average age of 72.5 ± 6.9 years (range, 65–83), were recruited from the community through poster advertisements. They did not have any history of stroke or other brain dysfunction, head and neck cancer or anatomic alterations in that area, speech or swallowing difficulties. Additionally, they were not taking any medication that could affect their swallowing function. This study was approved by the University Hospital Institutional Review Board and informed consent was obtained from every participant.

#### Test procedure

All participants were invited to the test room next to the TPA room and received a 10 min educational presentation of the sensory test before they participated in the experiments. Immediately after TPA, foods were provided in a random order and blinded manner, and they were asked to swallow 5 g of each test food at once without mastication. They rated each score about; 1) how much effort was required to swallow food, and 2) how much of the food remained in the pharynx after swallowing using a 9-point hedonic scale (1 = very less, 9 = very much).

### Statistical analysis

Statistical analyses were performed by using SPSS version 12.0 (SPSS, Inc., Chicago, IL, USA). The average value of hardness, adhesiveness and cohesiveness of each test food was obtained. Moreover, the average score of 18 older adults for two sensory tests was calculated. The Pearson correlation coefficient was used to evaluate the correlation between TPA and the sensory tests. The significance level was set at *p* < 0.05.

## Results

The average values of hardness, adhesiveness and cohesiveness for each food were as shown in Table 1. Foods that belonged to the same viscosity category showed different texture values. Whipped cream had low values of hardness and adhesiveness, and red bean paste showed high values in both. Boiled mashed pumpkin, potato and sweet potato were not high in hardness but in adhesiveness. Cohesiveness did not show distinctive features between foods. The scores of sensory tests were as shown in Table 1. The participants rated different scores respectively. Whipped cream was the easiest to swallow and mango pudding had the least feeling of sticking to the throat. Peanut butter was the hardest to swallow and had greatest sticky feeling.

**Table 1:** The average values of TPA and sensory tests for nine kinds of foods

Foods	TPA			Sensory tests	
	Hardness (N)	Adhesiveness (mJ)	Cohesiveness	Difficult to swallow	Sense of residue
Whipped cream	0.26 ± 0.03	0.87 ± 0.12	0.61 ± 0.04	1.39 ± 1.15	1.22 ± 0.73
Mayonnaise	0.63 ± 0.02	2.80 ± 0.10	0.87 ± 0.03	1.67 ± 1.33	1.67 ± 0.59
Soft tofu	1.59 ± 0.03	0.72 ± 0.08	0.51 ± 0.08	2.28 ± 1.74	1.28 ± 0.58
Mango pudding	4.06 ± 0.18	0.10 ± 0.10	0.64 ± 0.02	2.22 ± 2.24	1.00 ± 0.00
Boiled mashed pumpkin	2.12 ± 0.20	8.70 ± 0.40	0.76 ± 0.09	3.56 ± 2.20	2.72 ± 1.57
Boiled mashed potato	2.83 ± 0.09	13.27 ± 0.21	0.76 ± 0.03	4.83 ± 1.95	3.78 ± 1.80
Boiled mashed sweet potato	2.62 ± 0.43	9.77 ± 1.70	0.68 ± 0.08	5.94 ± 1.92	5.00 ± 1.91
Red bean paste	6.95 ± 0.28	27.55 ± 0.78	0.71 ± 0.08	6.61 ± 2.09	5.94 ± 2.44
Peanut butter	3.46 ± 0.32	18.30 ± 1.01	0.89 ± 0.04	7.61 ± 2.50	7.61 ± 2.70

**Fig. 2:**
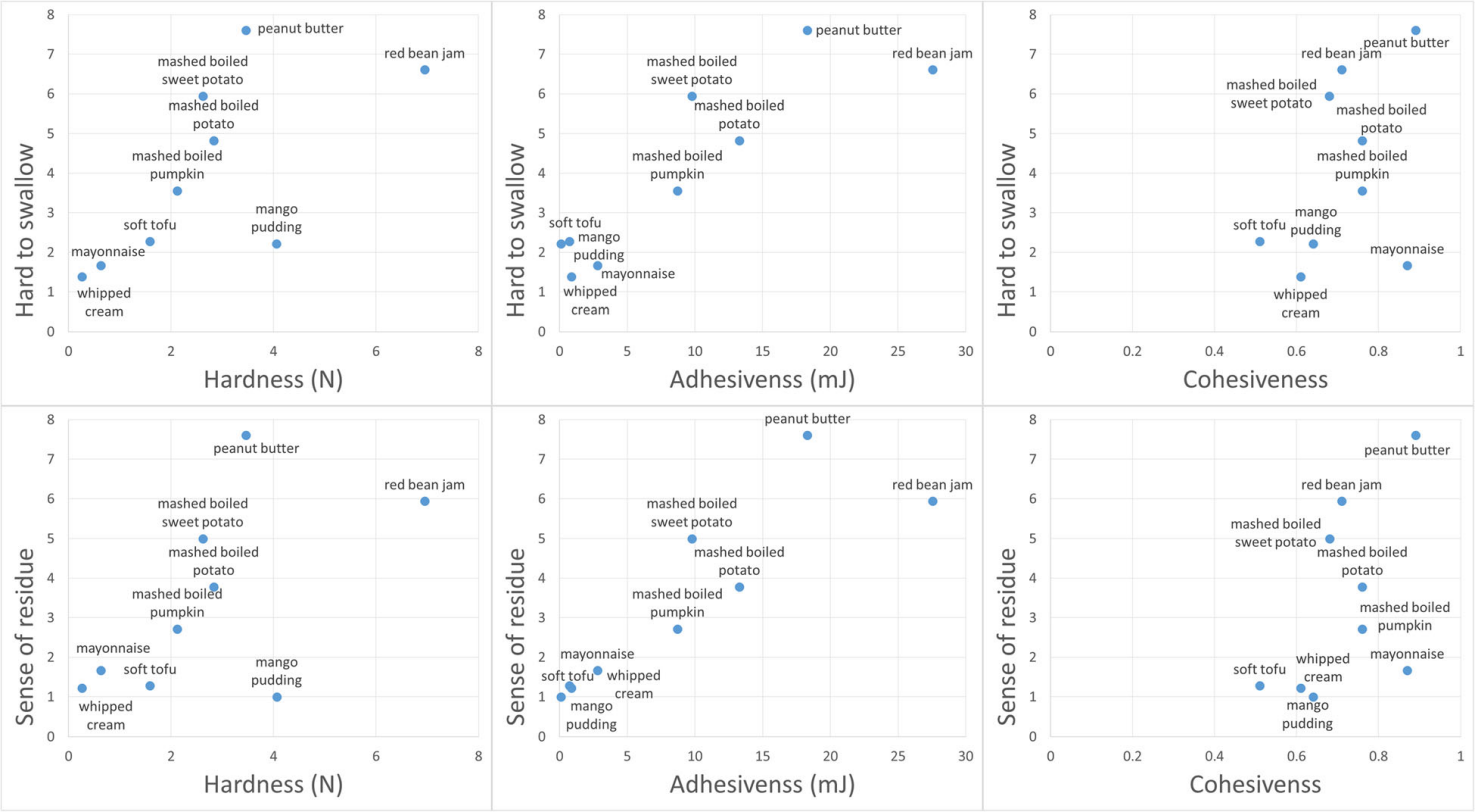
Scatter plots of sensory tests (difficult to swallow and sense of residue) with hardness, adhesiveness and cohesiveness. Only adhesiveness among the three properties was significantly correlated with both sensory tests. (r = 0.882, p = 0.002 for difficult to swallow, r = 0.879, *p* = 0.002 for sense of residue)

In the correlation test between TPA and sensory tests, only adhesiveness among three properties was significantly correlated with both sensory tests. (r = 0.882, *p* = 0.002 for difficult to swallow, r = 0.879, p = 0.002 for sense of residue) (Fig. 2).

## Discussion

It was found that among the three texture properties, adhesiveness was associated with the sensation of swallowing difficulty in the older adults. Adhesiveness is defined as the work necessary to overcome the attractive forces between the surface of the food and that of other materials with which the food comes into contact and calculated by measuring the negative area for the first bite, representing the work necessary to pull compressing probe away from food [13]. This definition might explain why adhesiveness is related to the difficulty of swallowing and sense of residue in the older adults.

To date, studies related to dysphagia diet have been conducted in terms of viscosity, [6, 10] and accordingly, classification of dysphagia diet has been mostly based on viscosity [15, 17]. Although viscosity is important, at the highest viscosity level (solid foods), further classification is primarily related to chewing (minced, grinded or soft etc.) and less related to pharyngeal swallowing. We assumed that other texture properties besides viscosity might be associated with pharyngeal swallowing difficulties in the older adults. We also found out the relevant possibility of adhesiveness.

Only one study mentioned the relation between food texture and dysphagia [18]. They investigated the association between the texture of semi-solid foods and fiberoptic endoscopic swallowing study findings in post-stroke dysphagic patients and suggested significant relation with cohesiveness according to residue deposition and gumminess according to aspiration. However, they had limitation about the use the different viscous foods restricted to stroke dysphagic patients and there was also a lack of explanation about the reason of their relations.

Motor function related to swallowing becomes increasingly dampened with age. Age-related anatomical changes in swallowing function include a decreased cross-sectional area of masticatory muscles (masseter and medial pterygoid), increased tongue atrophy and fatty infiltration and decreased diameter of tongue muscle fiber [19]. Age-related decrease in force, mobility and endurance is also definite in the lingual and pharyngeal muscle [20, 21]. For this reason, food texture affects swallowing in older people more.

Major limitation of this study is that sensory test is not sufficient to evaluate the effect of food texture properties on the swallowing performance. It might be a bit controversial to compare the TPA results with the sensorial test performed which is based on subjections. It would be better if more accurate functional data of swallowing physiology such as videofluoroscopic swallowing study were added. We have tried to reduce the likelihood of confounding factors. We only used foods that were commercially available at the market as finished products. We performed sensory tests immediately after the TPA test. Food was provided in a random and blinded order, and the participants swallowed food without chewing. Nevertheless, the problems of food homogeneity, mixing with saliva and temperature changes can make another limitation of this study.

## Conclusions

Adhesiveness was the most important property of semi-solid food requiring most efforts in pharyngeal swallow in the older adults. If we select and provide the food having low adhesiveness value in the same viscosity category, there might be a possibility to make it easier to swallow in the older adults.

## Supplementary Information

**Additional file 1.**

## Data Availability

All data generated or analyzed during this study are included in this published article.

## Abbreviations

IDDSI: International Dysphagia Diet Standardization Initiative
TPA: Texture profile analysis
