# Supplementary material for: Effects of texture properties of semi-solid food on the sensory test for pharyngeal swallowing effort in the older adults
Source: BMC Geriatr. 2020 Nov 23;20:493. doi: 10.1186/s12877-020-01890-4 (PMC7684931; doi:10.1186/s12877-020-01890-4)
Supplement: Supplementary file 1 — Additional file 1. [file 12877_2020_1890_MOESM1_ESM.pdf]

<Food 1>

1. How difficult is it to swallow this food?

1 2 3 4 5 6 7 8 9

Very easy Usually Very difficult

2. How much food remains in the pharynx after swallowing?

1 2 3 4 5 6 7 8 9

Very less Usually Very many
